# Effect of Milk-Feeding Frequency and Calcium Gluconate Supplementation on Growth, Health, and Reproductive and Metabolic Features of Holstein Heifers at a Rearing Farm

**DOI:** 10.3390/ani14091336

**Published:** 2024-04-29

**Authors:** Angel Revilla-Ruiz, Patricia Carulla, Aitor Fernandez-Novo, Eduardo de Mercado, Alejandro Pérez-Navarro, Raquel Patrón-Collantes, Francisco Sebastián, Sonia S. Pérez-Garnelo, Juan V. González-Martín, Fernando Estellés, Arantxa Villagrá, Susana Astiz

**Affiliations:** 1 Medicine and Surgery Department, Veterinary Faculty, Complutense University of Madrid (UCM), Avda. Pta. de Hierro s/n, 28040 Madrid, Spain; angelrevillaruiz@gmail.com (A.R.-R.); juanvi@vet.ucm.es (J.V.G.-M.); 2 Institute of Animal Science and Technology, Valencia Universitat Politècnica de València (UPV), Camino de Vera s/n, 46022 Valencia, Spain; patricia.carulla96@gmail.com (P.C.); feresbar@upv.es (F.E.); 3 Cowvet SL, País Valenciano Avenue 6, 46117 Betera-Valencia, Spain; pereznavarro.ale@gmail.com (A.P.-N.); fco.sebastian@gmail.com (F.S.); 4 Department of Veterinary Medicine, School of Biomedical and Health Sciences, Universidad Europea de Madrid, C/Tajo s/n, 28670 Villaviciosa de Odon, Spain; aitor.fernandez@universidadeuropea.es (A.F.-N.); raquel.patron@universidadeuropea.es (R.P.-C.); 5 Animal Reproduction Department, Instituto Nacional de Investigación y Tecnología Agraria y Alimentaria-Consejo Superior de Investigaciones Científicas (INIA-CSIC), Avda. Pta. Hierro s/n, 28040 Madrid, Spain; eduardo.mercado@inia.csic.es (E.d.M.); sgarnelo@inia.csic.es (S.S.P.-G.); 6 Centro de Tecnología Animal-Institut Valencià d’Investigacions Agràries (CITA-IVIA), Polígono La Esperanza 100, 12400 Segorbe, Spain; villagra_ara@gva.es

**Keywords:** heifers, first pregnancy, dairy, welfare, stress, metabolites

## Abstract

**Simple Summary:**

Female calves are the future, in terms of production, in every Holstein herd. Therefore, optimum rearing enhances productivity. Rearing dairy heifers involves important aspects of their early life that can affect their future efficiency. One of these key issues is nutrition management. Appropriate nutrition management impacts genetic performance, calf growth, and future productive and reproductive outcomes, as has been previously demonstrated in the literature. Traditionally, the recommended feeding strategies for dairy calves stipulate two daily milk replacement meals in a restricted diet. However, studies regarding feeding times point out different results in terms of efficiency, growth, and health in Holstein calves. We studied the effect of increasing feeding times (three instead of two per day) but with the same amount of milk replacer daily. Moreover, we included a supplementation of calcium gluconate to study its impact on growth, health, and productive and reproductive indexes in calves. We aimed to enhance welfare and digestive health early in life, expecting to induce positive medium- and long-term effects in the heifers. In fact, we observed a “catch-up” in the growth of calves since weaning, where supplementation with calcium gluconate appeared to reduce animal metabolic stress. We indeed observed a decreasing trend in the number of artificial inseminations per pregnancy, by 0.2 points, in heifers fed with milk replacer thrice daily. Finally, we confirmed significant correlations between early health and growth and reproductive efficiency. Although the medium- and long-term effects of these novel strategies were relatively weak, these management schemes seem to be promising for heifer rearing.

**Abstract:**

We compared the effects of milk-feeding in 288 Holstein calves (72 per group) which were fed twice (2F) or thrice (3F) daily, with or without the addition of hydrogenated fat-embedded calcium gluconate (G) supplemented in the starter food and in the daily diet up to the age of 9 months, on the calves’ metabolism, growth, health, and reproductive efficiency up to first pregnancy. The calves received 6 L of milk replacer (130 g/L) and had ad libitum access to water and textured calf starter with or without gluconate. Gluconate supplementation promoted a “catch-up” in growth in supplemented calves compared to their counterparts that did not receive gluconate. Gluconate appeared to reduce animal metabolic stress during key events, such as weaning and transfer into open-door pens, reducing fructosamine (352.61 vs. 303.06 in 3FG and 3F, respectively; *p* = 0.028) and urea (3F revealed the highest values compared with the other three groups: 19.06 for 3F vs. 13.9 (2F), 13.7 (2FG), and 14.3 (3FG), respectively, *p =* 0.002) from weaning onwards. The feeding of dairy calves with milk replacer three rather than two times per day tended to be associated with better health from weaning to 4 months old; parameters such as ultrasound lung score and calf health score improved over time (*p* < 0.001). Thrice-daily feeding with milk replacer tended to reduce the number of artificial inseminations per pregnancy in heifers by 0.2 points (*p* = 0.092). We confirmed significant correlations between early health and growth parameters and reproductive efficiency and a positive correlation between body weight and average daily weight gain and the thickness of the back fat layer in young heifers (*r* = 0.245; *p* < 0.0001; *r* = 0.214; *p* < 0.0001 respectively). Our study was conducted on a commercial farm with reasonably effective animal management, so baseline welfare was likely satisfactory.

## 1. Introduction

Rearing heifers is a key part of the dairy farm production system in terms of its significance for immediate and future economic sustainability. Rearing includes many daily decisions that determine heifer health, growth, and long-term survival and productivity. The most critical key performance indicator and therefore, objective [1], is age at first calving (AFC), which, to be optimal for productive performance, is set as maximum of 24 months old [2,3]. Achieving an AFC of 24 mo. results in the most cost savings [4,5]. Age at first calving is influenced by, among others, a heifer’s live weight and the age of puberty [6], which are directly affected by their health and welfare during their lifetime. In brief, heifers must grow adequately (and healthily) to achieve optimal results. The impact of disease during the heifer rearing period is significant, with losses associated with impaired future milk production performance and reproductive capacity as well as reduced survival [7]. Heifers with a history of diarrhea during the first month of life show lower chances of conception and calving than healthy heifers [8] and require more inseminations to become pregnant [9]. Lung consolidation (due to Bovine Respiratory Diseases, or BRDs) in the first 56 d of life reduces future milk production (525 kg less) during the first lactation [10,11]. Average daily weight gain and an incidence of BRDs early in life partially forecast future heifer productive success [12,13]. Finally, days of illness < 4 mo. old negatively affect first-lactation production levels [14]. Even factors that occur before birth or during the first 24 h of life can affect future performance. For example, heat stress suffered by fetal/newborn heifer calves show detrimental effects on the fertility and ovarian reserve of the offspring [15].

As seen above, the performance of rearing heifers is optimized when illness is avoided at their youngest age. Illness is considered the opposite of animal welfare. Thus, it seems plausible that improving welfare during the first stages of life may improve short-, medium-, and long-term health and productivity parameters. In recent years, there has been a shift in the rationale for animal welfare, from avoiding negative practices to promoting positive experiences, encouraging a “good life” [16,17]. In dairy cattle, calf rearing is one of the most challenging phases regarding health and animal welfare. Despite evidence that management affects how animals cope with stressors in their day-to-day lives, there is little research on how management can improve welfare early in life and how this improvement enhances performance. A recent systematic review identified feeding strategies and social management as key factors related to animal welfare in young heifers [18,19]. Specifically, among feeding strategies, milk replacer, colostrum, and weaning were identified as the main calf-rearing-related topics influencing animal welfare [18].

The recommended feeding strategies for dairy calves stipulate two daily meals in a restricted diet to promote starter feed intake and rumen development and reduce costs [20]. However, scientific evidence argues for increasing milk-feeding frequency, which is more aligned with natural behavior: calves feed several times a day when allowed [7,21]. More frequent feeding reduces time with pH values < 3 in the abomasum [22,23] and promotes abomasal emptying and the absorption of nutrients [24]. These situations can enhance abomasal and hindgut health. Moreover, a very recent study pointed out a helpful equation to determine calves’ body weight and solid feed intake [25], which is helpful for calculating feed intake needs and, therefore, maximizing hindgut health.

Intestinal health has also been enhanced with gluconic acid and its salt [26], avoiding the occurrence of a “leaky gut” [27]. In fact, gluconate supplementation has been shown to enhance the performance and health of piglets [28] and its inclusion before weaning enhanced the growth of heifers after the end of supplementation [29].

Therefore, enhancing digestive health in calves and heifers either with additives up to puberty or through increasing milk-feeding frequency are possible options which are likely to boost welfare. Nevertheless, the costs associated with such strategies are not trivial. Regarding increasing the number of milk feedings, one analysis suggests that the greatest costs on a dairy farm between calf birth to weaning are costs related to feeding [4]. Without automatic milk feeders, feeding calves on a dairy farm one additional meal per day can cost as much as USD 0.50 per kg of body weight gained [30]. Yet relatively few studies have examined the effects of feeding frequency on the short-, medium-, and long-term growth, health and productivity of dairy calves, and the studies that did came to contradictory conclusions [24,31,32,33,34]. Therefore, feeding frequency should be carefully and rigorously optimized, observing not only short-term effects, but long-term ones as well to evaluate the adequacy of these strategies for enhancing the welfare of young calves and heifers.

We hypothesize that the digestive health of dairy rearing calves and heifers would be positively influenced by thrice- vs. twice-daily milk-feeding and by gluconate supplementation up to 9 months of age, or by both. The expected effects of this enhancement in health would be improved growth, metabolism, and reproductive efficiency during the first pregnancy of dairy heifers. The results described here are part of a broader research project. The short-term effects of these strategies up to weaning are shared in a different scientific article. The aim in this part of the study was to explore the effects of the number of milk feedings per day (two vs. three times) combined with adding gluconate to the daily rations of calves up to the age of 9 months on a Spanish rearing farm on medium- and long-term blood metabolites, health, growth, and reproductive efficiency.

## 2. Materials and Methods

This study was approved by the Committee on Ethics in Animal Research at INIA-CSIC and by the Consellería de Agricultura Desarrollo Rural, Emergencia Climática y Transición Ecológica de la Generalitat Valenciana (code number 2023-VSC-PEA-0138). Study procedures complied with the Spanish Policy for Animal Protection (RD 53/2013) and European Union Directives 98/58/EC and 2010/63/UE.

### 2.1. Animals and Experimental Design

This study involved replacement heifers on a commercial rearing farm (Cowvet SL) in Valencia, Spain. The rearing farm has a capacity of 3000 animals, around 20% of which are in the preweaning phase. A total of 288 female Holstein dairy calves were enrolled in this study in 70 weekly batches and were included from 18 November 2020 to 19 December 2021, grouped into blocks by farm of origin and body weight. Within each block, animals were randomized into four experimental groups. Each group initially contained 72 animals. The groups were as follows: one group, 2FG, fed on milk twice a day and supplemented with gluconate (calcium gluconate embedded in hydrogenated fat in a percentage of 50%; Selko Lactibute, Trouw Nutrition, Amersfoort, The Netherlands), containing 72 calves; one group, 2F, fed on milk twice a day without the addition of gluconate (*n* = 72); one group, 3FG, fed on milk three times a day with the addition of gluconate (*n* = 72); and 3F, a group fed on milk three times a day without the addition of gluconate (*n* = 72). Milk-feeding was implemented at least during 15 days since the calves’ arrival at the rearing farm.

Only healthy calves between 5 and 38 days old were included. Calves were excluded if their rectal temperature was ≥39.7 °C or if they showed signs of disease (diarrhea, spontaneous coughing, or ocular or nasal discharge). Average age ± SD at enrollment was 16.9 ± 8.6 days (range 5 to 38 days old) and average age at weaning was 53.9 ± 4.20 days (range 44 to 66 days old).

Of the 288 calves enrolled in this study, six died (0–4 per group) between arrival and weaning; one calf from each group died between weaning and the end of gluconate supplementation. Another four calves had to be excluded from the twice-daily group without gluconate because there was an error in the administration of starter feed. The final number of calves included in the analysis up to end of gluconate supplementation (9 mo. of age) were 274 (2FG: 71; 2F: 63; 3FG: 70; and 3F: 70).

Upon arrival, calves were housed in pairs in pens with two polyethylene hutches (1.55 × 1.15 m^2^), a shared front courtyard (2 × 1.2 m^2^), and view to and contact with other calves. The hutches were in an open area and were bedded with sawdust, which was added twice weekly. One week after arrival, the animals were anesthetized with intramuscular 2% xylazine (0.3 mg/kg of body weight; Xilagesic, Laboratorios Calier SA, Barcelona, Spain) and provided with analgesia with meloxicam (0.5 mg/kg of body weight; Loxicom 20 mg/mL, Laboratorios Karizoo SA, Barcelona, Spain); then, disbudding was performed with heat cauterization. During the same week, the calves were vaccinated against parainfluenza-3 virus, bovine respiratory syncytial virus, and serotype A1 of Mannheimia haemolytica (Bovilis Bovipast RSP, MSD Animal Health, Carbajosa de la Sagrada, Spain). The animals were revaccinated at 2 months of age.

One week after weaning, the calves were transferred into group hutches (2 × 3 m^2^) featuring a forward courtyard (3 × 2 m^2^). At the age of 3.5 months, the calves were housed in shaded open-door group pens (12 animals per pen) featuring a feeding alley in the front. When the animals were 9 months old, gluconate supplementation ended and they were housed in loose housing barns in groups of 30 heifers until their transfer into the reproductive herd, which occurred depending on the availability of space but always when animals were older than 10 months and younger than 12 months. Here, the heifers were housed in groups of 48–60 animals in open-door, shaded housing barns with concrete flooring and self-locking head gates in the feeding alley. The roofed surface was bedded with sawdust.

### 2.2. Animal Feeding

After enrollment, animals were randomized into four groups, which were individually fed twice- or thrice-daily with milk replacer in teat bottles, with/without calcium gluconate in the starter feed, as explained above.

All calves had ad libitum access to water and textured calf starter feed containing 17.2% crude protein and 4% fat (NANTA, Madrid, Spain). The advised gluconate intake for milk-fed calves is 4.4 g/day/calf. The starter feed was therefore supplemented with a final concentration of 0.32% calcium gluconate (3.2 kg/ton) in the gluconate groups. Starter feed was distributed into individual troughs at 0.5 kg/calf/day for calves up to 20 d old, after which it was increased to 1.0 kg/calf/day until the age of 45 days, when it was increased to 1.5 kg/calf/day. Table 1 details the animal diets in this study. Ingredient details are included in Table S1 (Supplementary Material).

In each row, the two values refer to the groups without/with calcium gluconate supplementation. % refers to percent dry matter. See Section 2.2 for further description of the diet at different developmental stages. Abbreviations: ADF, acid-detergent fiber; AI, artificial insemination; CF, crude fiber; CP, crude protein; DM, dry matter; G-, fat-embedded calcium gluconate supplementation; H, humidity; ME, metabolizable energy; Milk FU, milk forage unit; NEL, net energy for lactation; NDF, neutral detergent fiber; NFC, non-fibrous carbohydrates; RDP, degradable protein; RUP, undegradable protein; TDN, total digestible nutrients.

All calves received the same diet of milk replacer without casein at a concentration of 130 g/L (0.78 kg DM/d, 6 L per day) with the following composition: 27% protein, 17% fat, 0.1% fiber, 1% calcium, 0.5% phosphorus, and all trace elements and vitamins recommended by the National Research Council [35]. Milk was manually administered using individual teat bottles at 09:00 and 14:00 h for groups fed twice daily, with an additional feeding at 18:00 h for groups fed three-times daily. During the week prior to weaning, when animals were a median of 45 days old (actual age 49.6 ± 6.3 days old), the daily volume of milk replacer was reduced progressively (“progressive weaning”). In the group fed twice a day, feedings were reduced to once daily in the morning during this week. In the calves fed three times a day, feeding was reduced to twice daily during the first three days of this week, and then calves were fed once daily in the morning. Only calf pairs that seemed to consume approximately >1000 g per calf of starter feed per day were weaned (actual weaning age 53.9 ± 4.2 days old).

After weaning, animals had ad libitum access to total mixed ration formulated according to the recommendations of the National Research Council ([35]; Table 1). The advised daily gluconate intake for heifers since weaning is 8.4 g/day/heifer. The ration for the gluconate-supplemented groups contained, therefore, 0.16% (1.6 kg/ton) calcium gluconate, since weaning up to the age of 9 months, added through the protein nucleus in the feed, at a concentration of 0.635% in the nucleus (expected daily intake 5.25 kg DM/day/heifer; 1.35 kg of protein nucleus per day and heifer). The calves received a “postweaning diet” up to the age of 4 months, which was starter feed containing 5% chopped straw (5.8 kg/animal/day, 5.12 kg DM/animal/day). Next, the animals received a total mixed “growth” ration up to the age of 7 months, at 15.35 kg/animal/day (7.11 kg DM/animal/day). Subsequently, the animals received a “prepuberty diet”, which was a wet total mixed ration at 16.55 kg/animal/day (8.17 kg DM/animal/day), until their transfer into the reproductive herd, where animals received an “insemination” diet of wet total mixed ration (17.65 kg/animal/day, 9.12 kg DM/animal/day). After they became pregnant, they received a “pregnancy diet” at 23.8 kg/animal/day (11.38 kg DM/animal/day) (Table 1 and Table S1).

### 2.3. Assessment of Metabolic Indices

**Figure 1:**
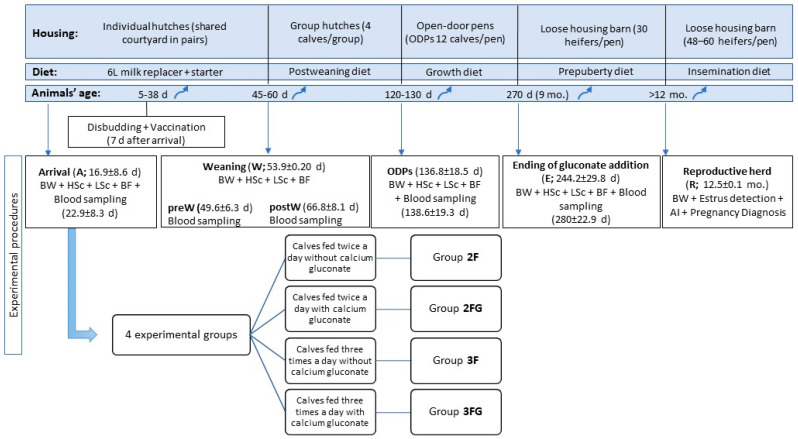
Schematic description of the animal groups and procedures in this study. Analyses were conducted at weaning, transfer into open-door pens (ODPs), end of gluconate supplementation (E), and transfer into the reproductive herd (R). Animal age at different time points is given in parentheses. AI, artificial insemination; BF, backfat thickness as measured by ultrasonography; BW, body weight; HSc, health score; LSc, ultrasound lung score; MR, milk replacer; pre-W, preweaning period; post-W, postweaning period.

A total of 140 animals were randomly selected at arrival to undergo blood sampling at various time points in the study (Figure 1). Blood was sampled into sterile plasma EDTA-tubes (BD-Plymouth. Franklin Lakes, NJ, USA) via puncture of the jugular vein in calves up to 4 mo. old and via puncture of the coccygeal vein in older animals. Blood was sampled between noon and 4: 00 h p.m. Blood sampling was performed a few days after other assessments in order to avoid overstressing the calves (Figure 1). Additionally, monthly blood samples were taken from heifers from an age of 3.5 months until the determination of puberty (Section 2.6).

Plasma was analyzed with a Konelab 20 clinical analyzer (Thermo Fisher Scientific, Waltham, MA, USA) according to the manufacturer’s instructions. The following metabolic markers and metabolites were determined (intra-assay and inter-assay variations, the coefficient of variation (CV, in %), and the correlation coefficient (*r*) are indicated in parentheses): glucose (98.5 ± 0.58, 92.5 ± 2.76, CV: 0.59%, and *r*: 0.995); fructosamine (217 ± 4.71, 197 ± 4.20, CV: 2.17%, and *r*: 0.985); lactate (13.8 ± 0.07, 14.3 ± 0.34, CV: 0.53%, and *r*: 0.998); β-hydroxybutyrate (2.3 ± 0.2, 2.4 ± 0.2, CV: 3.5%, and *r*: 0.999); non-esterified fatty acids (CV 0.89% and *r*: 0.997); triglycerides (109 ± 0.64, 111 ± 3.74, CV: 0.58%, and *r*: 0.998); total cholesterol (31.4 ± 0.42, 32.1 ± 0.92, CV: 1.35%, and *r*: 0.991); high- and low-density lipoprotein cholesterol (28.0 ± 0.25, 27.5 ± 1.26, CV: 0.89%, and *r*: 0.938); and urea (40.7 ± 0.88, 40.5 ± 1.19, CV: 2.16%, and *r*: 0.998).

### 2.4. Assessment of Growth

Growth was assessed in terms of body weight and backfat thickness at the animal ages indicated in Figure 1. Body weight was determined using a digital scale (EziWeigh7i; Tru-Test Livestock Management, County Cork, Ireland), which was calibrated daily before the first weighing at each measurement moment. Average daily weight gain was calculated for the different intervals: from weaning until transfer to open-door pens (ADW-W-ODP), from open-door pens until ending the gluconate supplementation (ADW-ODP-E), and from the end of gluconate supplementation until transfer into the reproductive herd (ADW-E-R).

Backfat was measured as described for adult cattle [36] with a linear 7.5-MHz probe (ProVetScan SR-2C, New Vetec S.L., León, Spain). After the unshaven skin area was rubbed with 70% alcohol, the probe was scanned along an imaginary line between the tuber coxae (hook) and tuber ischia (pin) at the sacral region; the skin was held lightly and orthogonally to the interface of fat and muscle during scanning. The thickness of the subcutaneous fat layer was measured in mm on appropriate frozen images. Two farm veterinarians performed all these measurements.

### 2.5. Assessment of Health

We scored health using a scale adapted from a previously described one ([37]; Table 2). Farm staff screened the animals daily for disease, which was initially detected as reduced feed intake or signs of apathy. Animals suspected of being sick were examined by farm veterinarians. Scour was diagnosed based on the appearance of loose feces (fecal score ≥ 2) and signs of dehydration as well as reduced or no intake. Bovine respiratory disease (BRD) was diagnosed if rectal temperature exceeded 39.7 °C in the presence of at least two of the following: apathy, ocular or nasal discharge, cough, dyspnea, or accelerated breathing.

Lung health was assessed in the animals using an ultrasound-based lung score. The abovementioned linear probe was used as described [38] over the right lung from the 10th intercostal space cranial to the first intercostal space and over the left lung from the 10th intercostal space cranial to the second intercostal space. Before scanning, the unshaven skin was rubbed with 70% isopropyl alcohol. Animals were assigned one of the following scores: 0, normal aerated lung; 1, diffuse comet-tail artifacts without consolidation; 2, lobular or patchy pneumonia; 3, pneumonia affecting only one lobe; 4, pneumonia affecting two lobes; or 5, pneumonia affecting at least three lobes.

### 2.6. Assessment of Reproductive Ability and Performance

Levels of progesterone in plasma were assayed monthly from the age of 3.5 months with an enzyme-linked immunosorbent assay (ELISA; Demeditec Diagnostics, Kiel-Wellsee, Germany). Assay sensitivity was 0.045 ng/mL and the manufacturer-specified intra-assay variation coefficient was 5%. Age at puberty was defined as 15 days before the age when the level was at least 0.8 ng/mL. Alternatively, puberty was determined based on typical estrus behavior determined by an increase of 30% in daily activity based on data collected by a monitoring collar (SenseHub, Netanya, Israel) and analyzed by Allflex Livestock Intelligence software associated software Pro v.20.3.6.0 (MSD Animal Health Intelligence, Merck & Co., Rahway, NJ, USA).

Heifers were artificially inseminated with sexed semen from four bulls. Two farm veterinarians performed all inseminations in this study, and at days 28–35 later, they diagnosed pregnancies using the abovementioned ultrasound device. Pregnancy was confirmed by ultrasonography on days 50–56 and again on days 100–113 after insemination. If they did not become pregnant, heifers were re-inseminated up to four times after estrus observation; if this proved unsuccessful, heifers were allowed to mate naturally with the resident bull.

### 2.7. Statistical Analyses

Data were analyzed using SPSS 29 (IBM, Armonk, NY, USA), and results associated with *p* < 0.05 were considered significant. Continuous data were assessed for normality using the Kolmogorov–Smirnov test on residuals. Data for backfat thickness, calf health score, ultrasound lung score, and plasma lactate showed skew and were therefore reported as medians (interquartile ranges). Otherwise, data were reported as means and standard deviations.

**Figure 2:**
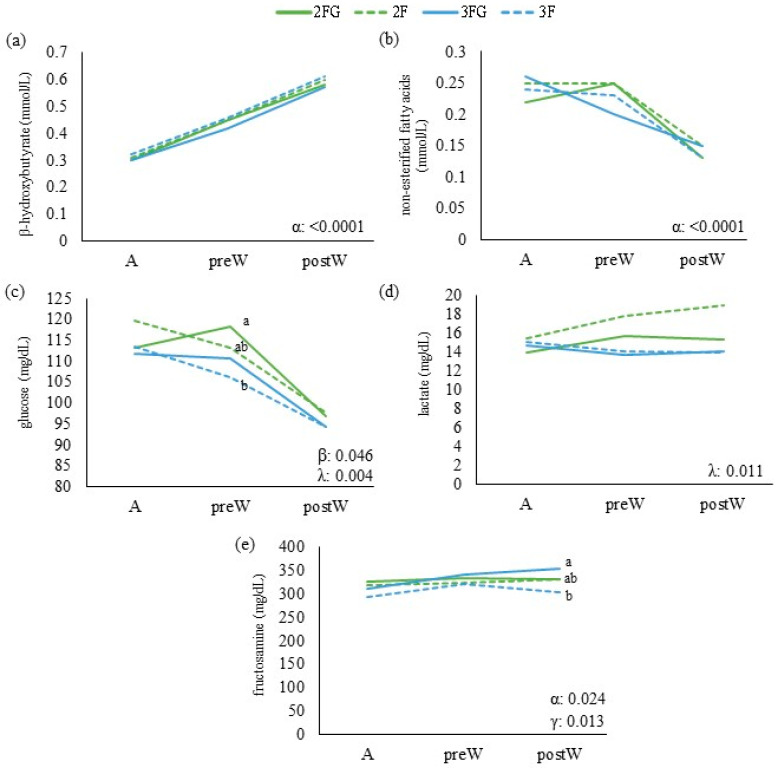
Energy-related metabolites of dairy calves at weaning (W), at the moment of transfer into open-door pens (ODPs), and at the end of gluconate supplementation (E): (**a**) β-hydroxybutyrate, (**b**) non-esterified fatty acids, (**c**) glucose, (**d**) lactate, and (**e**) fructosamine. Calves were randomized to be fed twice daily (2F) or three times a day (3F) with milk replacer, in both cases with ad libitum access to solid feed with or without the addition of calcium gluconate (G). Data are mean ± SD. a and b, significant differences among groups; α, significance of the effect of time in a mixed model; γ, significance of the effect of gluconate supplementation in a mixed model; β, significance of the effect of the interaction between time and gluconate; λ, significance of the effect of feeding times.

**Figure 3:**
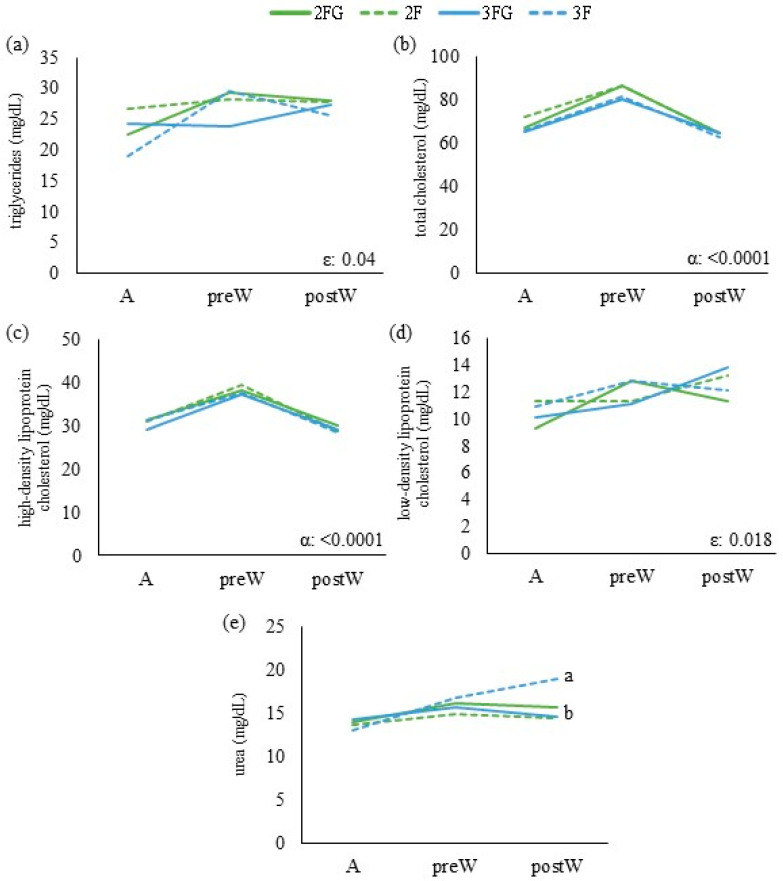
Lipid-related metabolites and urea of dairy calves at weaning (W), at the moment of transfer into open-door pens (ODPs), and at the end of gluconate supplementation (E): (**a**) triglycerides, (**b**) total cholesterol, (**c**) high-density lipoprotein cholesterol, (**d**) low-density lipoprotein cholesterol, and (**e**) urea. Data are mean ± SD. a, b, significant differences among groups; α, significance of the effect of time in a mixed model; ε, significance of the effect of the triple interaction of time, gluconate, and feeding frequency.

**Figure 4:**
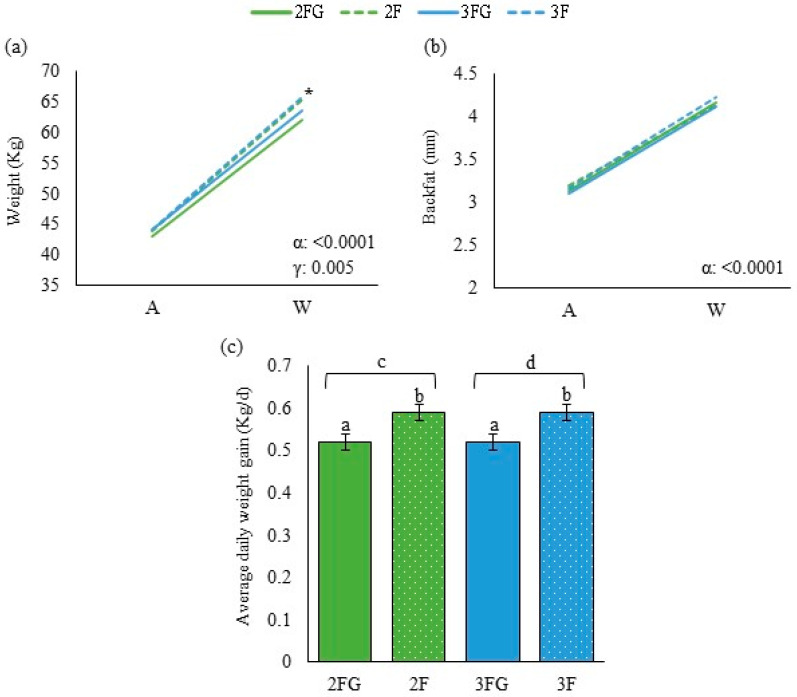
Growth parameters of dairy calves at the moment of weaning (W), at the moment of transfer into open-door pens (ODPs), at the end of gluconate supplementation (E), and at transfer into the reproductive herd (R) only in the case of body weight: (**a**) body weight in kg, (**b**) backfat thickness as measured by ultrasonography, and (**c**) average daily weight gain in three intervals. Calves were randomized to be fed twice daily (2F) or three times a day (3F) with milk replacer, in both cases with ad libitum access to solid feed with or without the addition of calcium gluconate (G). Data are mean ± SD. W-ODP: average daily weight gain from weaning to ODP transfer; ODP-E: average daily weight gain from ODP transfer to moment E; E-R: average daily weight gain from moment E to moment R. * in panel a indicates a significant difference between group 2FG and either group 2F or 3F at weaning (*p* < 0.05). a, b, c, d, significant differences among groups. α indicates a significant effect of time; γ, indicates a significant effect of gluconate supplementation.

Inter-group differences in continuous variables (energy-related metabolites, lipid-related metabolites, growth parameters, calf health and ultrasound lung scores) at a given time point were assessed for significance using univariate ANOVA and the Bonferroni test if the data were normally distributed. Otherwise, differences were assessed using the Kruskal–Wallis or Mann–Whitney U tests for independent samples. The results of these analyses are shown in Figure 1, Figure 2, Figure 3 and Figure 4 with letters a, b, and c to highlight differences among the groups at these single time points. Potential pairwise relationships were assessed using the Pearson test. Inter-group differences in categorical variables at a given time point were assessed using the chi-squared test.

For variables measured repeatedly (i.e., energy-related metabolites, lipid-related metabolites, growth parameters, calf health and ultrasound lung scores), we separately analyzed data from two periods between which animal diet or production circumstances differed substantially: one period extended from weaning to either the end of gluconate supplementation or transfer into the reproductive herd, and the second one from puberty to first pregnancy.

Generalized linear mixed models for the mixed-effects model (Mixed Model Analysis by SPSS^®^ v.29 Software) were implemented with Greenhouse–Geisser correction to assess variations over time in body weight, backfat thickness, calf health score, ultrasound lung score, and metabolic indices. Models included gluconate supplementation and feeding frequency as fixed effects, and “block” (see Section 2.1) as a random effect. The potential interactions of feeding frequency and gluconate supplementation with each other and with time were explored. Mixed models were used to calculate changes in variables across weaning, transfer into open-door pens, end of gluconate supplementation and, in some cases, transfer into the reproductive herd. These models included body weight at weaning as a covariate because it differed among the four groups at that moment of the study (weaning). To analyze the second period, mixed models were also used to compare, across the four groups, animal age at the following events: puberty, transfer into the reproductive herd, first artificial insemination, and first pregnancy. This model included body weight at weaning as a covariate.

The results of these analyses of repeated measures over time are shown in Figure 1, Figure 2, Figure 3 and Figure 4 with Greek letters, with α indicating a significant effect of time; ε, significance of the effect of the triple interaction of time, gluconate, and feeding frequency; γ, a significant effect of gluconate supplementation; β, significance of the effect of the interaction between time and gluconate; and λ, significance of the effect of feeding times.

**Figure 5:**
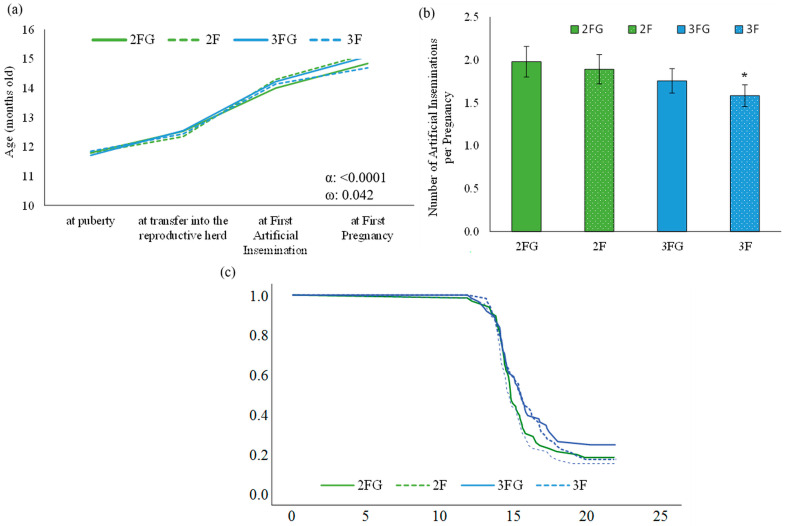
Reproductive parameters of dairy heifers: (**a**) age at different moments of reproductive life, (**b**) artificial inseminations per pregnancy for first pregnancy, and (**c**) survival curve showing the hazard ratio associated with age at first pregnancy. Heifers were randomized to be fed twice daily (2F) or three times a day (3F) with milk replacer during lactation, in both cases with ad libitum access to solid feed with or without the addition of calcium gluconate (G). Data are mean ± SD. * in panel (**b**) indicates a tendency of difference between group 3FG and either group 2F, 3F and 2FG (*p* = 0.092).

Incidence of mortality and pregnancy was analyzed using Kaplan–Meier curves and Cox proportional hazards regression, with differences between the curves of age at pregnancy being assessed for significance using the Mantel–Cox test; the results of these analyses are shown in Figure 5. Body weight at weaning was treated as a covariate and “blocks” were treated as a random factor.

## 3. Results

### 3.1. Metabolic Indices

From weaning until the end of gluconate supplementation, the levels of β-hydroxybutyrate and non-esterified fatty acids did not differ significantly across the four groups or over time (Figure 2). When the animals were transferred to open-door pens, lactate levels were significantly lower in animals that received gluconate than in those that did not, and they were lower among animals fed thrice a day than among animals fed twice that received gluconate. From weaning to transfer into open-door pens, the levels of fructosamine decreased in animals that received gluconate but increased in those that did not. Subsequently, animals that received gluconate showed an increase in fructosamine if they were fed twice daily but a decrease if fed three times. We did not observe any systematic effects of feeding frequency or gluconate on the levels of lipid metabolic indices (Figure 3).

### 3.2. Growth Parameters

Although body weight at weaning was significantly lower for the two groups that received gluconate supplementation than for the other two groups, we found that weight, average daily weight gain, and backfat thickness did not differ significantly across the four groups at later time points (Figure 4).

Backfat thickness at transfer into open-door pens correlated with body weight at the end of gluconate supplementation (*r* = 0.245; *p* < 0.0001) and with body weight at transfer into the reproductive herd (*r* = 0.214; *p* < 0.0001). Backfat thickness at the end of gluconate supplementation correlated with body weight at that time point (*r* = 0.290; *p* < 0.0001) and with body weight at transfer into the reproductive herd (*r* = 0.210; *p* < 0.0001). Backfat thickness at transfer into open-door pens correlated negatively with the level of β-hydroxybutyrate at the same time point (*r* = −0.225; *p* = 0.01) and with blood glucose levels at the end of gluconate supplementation (*r* = −0.216; *p* = 0.014). Backfat thickness at the end of gluconate supplementation correlated negatively with the levels of lactate at transfer into the reproductive herd (*r* = −0.210; *p* = 0.0184) but positively with the levels of triglycerides at that point (*r* = 0.210; *p* = 0.018).

Body weight at transfer into open-door pens correlated positively with the levels of total cholesterol (*r* = 0.527; *p* < 0.0001) and high-density lipoprotein at the same time point (*r* = 0.245; *p* = 0.005), negatively with the levels of β hydroxybutyrate (*r* = −0.339; *p* < 0.0001) and triglycerides (*r* = −0.226; *p* = 0.011) at the end of gluconate supplementation, and positively with the levels of non-esterified fatty acids at the end of gluconate supplementation (*r* = 0.340; *p* < 0.0001).

### 3.3. Health Parameters

Both ultrasound lung scores and calf health scores improved over time, and no significant differences in improvement were observed across the four groups (Table 3).

Two calves died (one from the twice-daily group with added gluconate and one from the group fed three times a day with added gluconate) from weaning to transfer into the reproductive herd. The global rate of mortality (10/288; 3.5%) from arrival to the age of 305 days did not differ significantly across the four groups (2FG: 1/72; 2F: 5/72; 3FG: 2/72, and 3F:2/72; *p* = 0.292), and it ranged from two to five animals per group. Among the calves that died, those that had not received gluconate were significantly younger than those that had (4.2 vs. 130 days old; *p* = 0.023). In the Cox regression study, weight at arrival, weight at weaning, and animal block were not significant predictors of mortality from arrival until reproductive age.

Backfat thickness at the end of gluconate supplementation correlated negatively with health score at ending the gluconate supplementation (*r* = −0.400; *p* < 0.0001). At transfer into open-door pens and at the end of gluconate supplementation, ultrasound lung score positively correlated with health score (*r* = 0.612; *p* < 0.0001), while both scores correlated negatively with body weight in three groups (*r* = −0.311; *p* < 0.0001 for health score and *r* = −0.303; *p* < 0.0001). Several correlations were observed between lung scores and metabolic indices that differed among the four groups but not in any systematic way that might suggest the influence of feeding frequency or gluconate supplementation on these parameters.

Heifers were randomized to be fed on milk twice (2F) or thrice (3F) daily, with ad libitum access to solid feed with or without the addition of calcium gluconate (G) up to the end of supplementation (9 mo. old). *p*-values for comparisons and interactions for the described intervals are from mixed models. Statistical significance was highlighted in bold.

### 3.4. Reproductive Parameters

The four groups did not differ significantly in age at puberty (11.8 ± 0.1 months across all animals), level of progesterone (3.2 ± 0.5 ng/mL), age at first artificial insemination (14.2 ± 0.06 months), age at first pregnancy (14.9 0.1 months), or number of inseminations per pregnancy (1.8 ± 0.08; Figure 5). However, pregnancy was significantly delayed among animals that had been fed three times a day with the addition of gluconate relative to pregnancy in the other groups (*p* = 0.042 in the mixed model), yet neither survival analysis of age at first pregnancy nor Cox regression detected significant differences among the groups (Figure 5). The number of artificial inseminations per pregnancy was 1.8 ± 0.08 inseminations across the four groups; 1.9 ± 1.2 for heifers fed twice daily with the addition of gluconate; 1.9 ± 1.2 for those fed twice daily with no supplementation; 1.8 ± 1.1 for heifers fed three times a day with the addition of gluconate; and 1.6 ± 1.0 for heifers fed three times a day with no supplementation. There was no statistical significance among the four groups (Figure 5). The number of artificial inseminations per pregnancy tended to be lower in the three-times-daily fed group than in the twice-daily group (1.9 ± 1.2 and 1.7 ± 1.04 artificial inseminations per pregnancy, respectively; *p* = 0.092; Figure 5).

Parameters related to health correlated significantly with reproductive efficiency. Specifically, age at first pregnancy correlated positively with age at onset of BRD (*r* = 0.538; *p* < 0.0001) and lung score at arrival (*r* = 0.146; *p* = 0.038), while health score at arrival (*r* = 0.225; *p* < 0.0001) and temperature at arrival (*r* = 0.256; *p* < 0.0001) correlated positively with age at puberty.

Weight at weaning correlated negatively with age at puberty (*r* = −0.182; *p* = 0.004), while average daily weight gain from arrival to weaning correlated negatively with age at first pregnancy (*r* = −0.160; *p* = 0.027).

## 4. Discussion

The aim of this study was to explore the effects of the number of milk feedings per day (two vs. three times) combined with the addition of gluconate to the daily ration of calves up to the age of 9 months on a Spanish rearing farm on the calves’ medium- and long-term blood metabolism, health, growth, and reproductive efficiency. Our analysis of calves on a commercial rearing dairy farm from when they were milk-fed until their first pregnancy suggests that gluconate supplementation promoted a “catch-up” in growth in calves with lower weight at weaning and led to differences in metabolic markers, dampening the effects of stress on metabolism. We confirmed significant correlations of early health and growth parameters with reproductive efficiency across the four groups, using the thickness of the backfat layer in heifers and validating it as an easily measurable index for assessing body condition in young dairy stock. Finally, a tendency to improve reproductive efficiency in terms of AI/pregnancy was observed as a result of the three-times-a-day milk-feeding strategy.

A decline in blood glucose was particularly noticeable from the calves’ transfer into open-door pens (average age 120–130 d old) until 9 months of age, reflecting the shift to ruminant adulthood [39]. During the development of our heifers, the levels of non-esterified fatty acids increased from weaning onwards, which is also consistent with the shift to a ruminant lifestyle [40]. We did not detect obvious relationships of levels of low-density lipoprotein with feeding frequency, gluconate supplementation, or time. Little is known about the low-density lipoprotein concentration in the blood of milk-fed Holstein calves and how they respond to different types of animal management or about how they change during growth. Future work should explore these questions [41] and whether they involve the “phosphatase and tensin homolog” [42]. We observed the highest level of urea at weaning in the thrice-daily group that was not supplemented with gluconate. Urea increases with high protein intake as rumen-degradable protein is broken down into ammonia and detoxified to urea. During periods of energy restriction, however, the increased compensatory catabolism of body protein also results in increased urea values [43]. Thus, at that stage in our study, increases in urea were probably driven mainly by the shift to a higher intake of dry feed, which increased protein and anabolic metabolism in all heifers [44,45,46].

As part of this study, we conducted what appears to be the first longitudinal analysis of backfat thickness in milk-fed dairy calves until puberty. Our data demonstrate a significant positive correlation of backfat thickness with body weight and average daily weight gain during this developmental period, suggesting that this ultrasound-based index can help assess body condition in calves and young heifers. We suggest that backfat thickness can be a complementary index to body weight in dairy calves and heifers, as already demonstrated in adult cows [47].

Our heifers showed adequate growth curves, similar to those described in other commercial farms in Europe [48,49,50,51]. The observation that calves on gluconate supplementation, despite having a lower body weight at weaning, nevertheless achieved similar growth as animals that did not receive gluconate supplementation implies that animals on gluconate experienced compensatory growth after weaning. Whether this resulted from a specific effect of gluconate or other compensatory processes remains to be clarified. We speculate that this compensatory growth may involve alterations in nutrient absorption [52] or the microbiome [53] or reflect the ability of calcium gluconate to prevent leaky gut, especially under stressful situations such as the hot season [54], which should be explored in future works. This compensatory growth could also be a consequence of the effect of gluconate on health and (numerically) on mortality.

Moreover, metabolic changes which alter energy and nitrogen metabolism influence growth rates [55]. Our results link gluconate supplementation to higher levels of fructosamine. Fructosamine, an energetic marker that provides information on long-term energy reserves, should decrease slightly with age but may increase in the presence of chronic stress [56,57], similar to glucose. Gluconate supplementation increasing glucose availability explains the higher levels of fructosamine observed during the week after weaning (no stressful situation). On the other hand, at the end of gluconate supplementation, the twice-daily group that had not received gluconate supplementation showed higher levels of fructosamine, indicating that these animals were less able to adapt to the stresses of transfer into open-door pens or later life events. Heifers in group 2FG showed lower levels of high-density lipoprotein than those that had not received gluconate. High-density lipoprotein has anti-inflammatory and anti-oxidant properties [58], and higher levels may reflect a reaction to stress. Indeed, high-density lipoprotein levels increased across all four groups during the stressful situation of transfer into open-door pens. All this may provide evidence that gluconate can help mitigate metabolic stress in heifers, with differences in especially stressful situations. In fact, other authors observed a significant positive effect of certain nutritional strategies in milk-fed calves only during stressful situations such as the hot season [24].

Even though more frequent milk-feeding did not significantly improve medium- and long-term animal health or growth in our study, several observations lead us to conclude that thrice-daily feeding may be preferable to twice-daily feeding and should therefore be explored further. Indeed, the twice-daily group showed marginally higher levels of lactate at weaning than the thrice-daily group; weaning is a stressful event that can trigger increases in lactate [59] and fibrinogen [60]. Therefore, thrice-daily feeding may have led to better metabolic status that made calves more resilient to new, stressful situations like weaning.

Neither feeding frequency nor gluconate supplementation significantly or clearly affected any of the reproductive indices that we examined, and the values in our study were similar to those reported for heifers on commercial farms in other European countries and USA [20,61,62] which have been associated with efficient rearing [20,63,64]. Consistently with this, we found that levels of total cholesterol as well as low- and high-density lipoprotein were within physiological ranges for prepubertal calves and pubertal heifers [65,66,67], suggesting healthy follicle growth and good reproductive performance in the future [68].

However, we observed a significant interaction of gluconate with time, influencing the age at first pregnancy. Heifers from the thrice-daily group on gluconate were marginally older at pregnancy than pregnant heifers in the other groups, which may be attributed to the fact that the thrice-daily group on gluconate also showed the lowest growth between arrival and weaning. This observation is consistent with the idea that adversity early in life can harm the reproductive efficiency of heifers [12,13]. On the other hand, thrice-daily feeding with milk replacer was associated with a reduction of 0.2 in the number of artificial inseminations per pregnancy in heifers. Supporting evidence on the long-term effects of early management strategies during calves’ milk-feeding [6] also comes from our observations on correlations of calf health score at arrival and daily average weight gain from arrival to weaning with age at puberty, age at first artificial insemination, and the number of inseminations per pregnancy.

We found that age at onset of bovine respiratory disease correlated positively with age at first pregnancy and with the number of artificial inseminations per pregnancy. This indicates worse reproductive performance among animals that experienced respiratory disease later but always before 180 days of age. This may reflect that disease onset closer to puberty can disrupt reproductive function more severely [69,70,71]. It may also reflect that disease in older animals follows a more severe course [70,72,73]. Unfortunately, we did not collect data on the severity of the disease in our animals, highlighting the need for future works.

Feeding strategies on farms should be implemented according to the available human resources and farmer commitment. Increasing feeding times from two to three may result in better health and reproductive performance. Moreover, adding gluconate seems to reduce stress biomarkers at stressful events. However, these strategies increase costs, and their financial impact on farm economics should be further studied.

Despite our relatively large sample, we may have failed to observe greater medium- and long-term benefits of thrice-daily feeding and gluconate supplementation because the general health and development conditions on the commercial farm were already high [74,75]. One indication of effective animal management on our farm was that at all time points, the levels of numerous metabolic indices remained within physiological ranges [67,68,69] and an adequate growth rhythm was observed in our heifers [48]. Moreover, a narrow health follow-up was performed on each individual heifer, and more effective health screening among calves and heifers is associated with higher rates of disease diagnosis and better cure rates [76]. Our study was conducted on a commercial farm with effective animal management, so baseline welfare was probably fairly good. This may help explain why the potential benefits that we observed for thrice-daily feeding and gluconate supplementation were relatively weak in the medium and short terms, due to the low potency of this study to identify small differences as significant.

## 5. Conclusions

The novel nutritional strategies implemented in this study in dairy calves and heifers at early ages partly improved their medium- and long-term efficiency. On this commercial farm in Spain, feeding dairy calves with milk replacer three rather than two times per day seemed to be associated with better health but not better growth or reproductive performance, although it did tend to reduce the number of inseminations for first pregnancy. Nevertheless, it was associated with lower metabolic stress based on various metabolic indicators. Calcium gluconate was linked to a “catch up” in growth after weaning and appeared to reduce animal metabolic stress at key events such as weaning and transfer into open-door pens, reducing lactate, fructosamine, and urea from weaning onwards.

## Figures and Tables

**Table 1:** Values of diets employed during this study.

Item	Starter Feed	Postweaning Feed	Growth Feed	Prepuberty Feed	AI Feed	Pregnant Feed
H, %	11.3/11.3	11.7/11.7	53.7/53.6	50.6/50.6	48.3	52.2
DM, %	88.7/88.7	88.3/88.3	46.3/46.4	49.4/49.4	51.7	47.8
Forage, %		20.6/20.6	27.2/27.2	32.3/32.3	46.3	49.5
ME, Mcal/kg	3113/3104	2783/2778	2462/2465	2400/2399	2173	2092
NEL, Mcal/kg	2.0/2.0	1.8/1.8	1.6/1.5	1.5/1.5	1.3	1.3
CP, %	19.0/19.0	16.9/16.8	16.1/16.1	15.1/15.1	14.6	14.0
RDP, %	11.0/10.9	8.9/8.9	9.9/9.9	9.3/9.3	8.4	8.3
RUP, %	8.1/8.1	8.0/8.0	6.2/6.2	5.8/5.8	6.2	5.7
CF, %	8.1/8.1	13.5/13.5	18.4/18.4	19.8/19.8	25.9	27.4
ADF, %	10.8/10.8	20.0/20.0	27.3/27.3	28.8/28.8	35.9	38.0
NDF, %	20.9/20.8	35.5/35.4	44.2/44.2	46.4/46.4	58.9	62.2
Starch	40.1/40.0	33.0/32.9	18.8/18.8	18.9/18.9	7.2	4.6
Sugar	4.6/4.6	5.9/6.0	3.7/3.7	3.4/3.4	4.0	3.3
NFC, %	51.0/50.9	37.6/37.5	24.3/24.3	23.9/23.9	13.3	10.5
Fat, %	3.3/3.3	4.2/4.2	7.5/7.5	6.9/6.9	5.8	5.8
Ash, %	5.8/6.0	5.9/6.0	8.3/8.4	8.1/8.1	7.8	7.9
TDN, %	80.0/79.8	71.7/71.6	67.2/67.2	66.1/66.2	60.5	59.2
Ca, %	0.71/0.72	0.91/0.92	1.12/1.13	1.12/1.13	0.9	0.9
P, %	0.4/0.4	0.5/0.5	0.4/0.4	0.4/0.4	0.4	0.3
Milk FU		1.0/1.0	0.9/0.9	0.9/0.9	0.8	0.7
Na, %	0.2/0.2	0.1/0.1	0.3/0.3	0.3/0.3	0.3	0.3

**Table 2:** Health scoring scale in this study.

Parameter	Score 0	Score 1	Score 2	Score 3
Rectal temperature, °C	37.8–38.2	38.3–38.8	38.9–39.4	≥39.5
Cough	None	Single cough	Induced or spontaneous occasional cough	Repeated spontaneous cough
Nasal discharge	Normal	Minimal, unilateral, cloudy	Bilateral mucus	Copious mucopurulent
Ear posture	Normal	Ear flick	Unilateral drop	Head tilt
Ocular discharge	Normal	Minimal	Moderate	Heavy
Feces	Normal consistency	Semiformed, pasty	Loose, but stays on top of bedding	Watery, sifts through bedding

**Table 3:** Calf health scores and ultrasound lung scores of heifers at the following moments considered in this study: transfer into open-door pens and end of gluconate supplementation.

		Values at Weaning	Values at Open-Door Pen Transfer	*p* Values from Weaning to Open-Door Pen Transfer	Values at End of Gluconate Supplementation	*p* Values from Open-Door Pen Transfer to End of Gluconate Supplementation
Ultrasound lung score (points)	2FG	2.0 ± 0.54	1.87 ± 0.56	2F vs. 3F: 0.073 G: 0.977 2F vs. 3F × G: 0.423 2F vs. 3F × G × time: 0.690 **time: 0.032**	1.63 ± 0.64	2F vs. 3F: 0.973 G: 0.637 2F vs. 3F × G: 0.904 2F vs. 3F × G × time: 0.690 **time: <0.001**
	2F	1.86 ± 0.53	1.78 ± 0.52		1.59 ± 0.56	
	3FG	1.82 ± 0.61	1.9 ± 0.54		1.6 ± 0.55	
	3F	1.76 ± 0.58	1.8 ± 0.58		1.6 ± 0.62	
Health score (points)	2FG	1.86 ± 0.52	1.77 ± 0.51	2F vs. 3F: 0.081 G: 0.978 2F vs. 3F × G: 0.917 2F vs. 3F × G × time: 0.985 **time: 0.003**	1.41 ± 0.52	2F vs. 3F: 0.960 G: 0.622 2F vs. 3F × G: 0.864 2F vs. 3F × G × time: 0.985 **time: <0.001**
	2F	1.81 ± 0.53	1.78 ± 0.49		1.35 ± 0.54	
	3FG	1.68 ± 0.62	1.83 ± 0.45		1.43 ± 0.53	
	3F	1.65 ± 0.51	1.84 ± 0.47		1.39 ± 0.55	

## Data Availability

The raw data supporting the conclusions of this article will be made available by the authors on request.

## Supplementary Materials

The following supporting information can be downloaded at: https://www.mdpi.com/article/10.3390/ani14091336/s1, Table S1: Ingredient composition of diets.
