# Supplementary material for: Effect of Milk-Feeding Frequency and Calcium Gluconate Supplementation on Growth, Health, and Reproductive and Metabolic Features of Holstein Heifers at a Rearing Farm
Source: Animals (Basel). 2024 Apr 29;14(9):1336. doi: 10.3390/ani14091336 (PMC11083690; doi:10.3390/ani14091336)
Supplement: Supplementary file 1 [file animals-14-01336-s001.zip › animals-2932274-supplementary.pdf]

Table S1. Ingredient composition of diets.

Ingredient, % of diet DM[1]	Starter Feed	Post-weaning Feed	Growth Feed	Pre-puberty Feed	AI Feed
Wet brewers' grains			35.91	33.5	52.36
Straw			13.17	16.75	20.42
Wet corn gluten feed					19.9
Rapeseed	4.27	16.93	1.38	1.38	2.62
Cocoa hulls					2.62
Additive	0.26	0.14	0.05	0.04	1.05
Carbonate	1.19	1.18			0.26
Orange pulp			31.92	29.78	
Straw		20.31			
Corn	35.76	44.01	9.58	10.61	
Gluten feed			5.19	5.19	
Soybean meal 47%	12.92		0.81	0.81	
Oat hulls			0.61	0.61	
Corn DDG[2]	8.15	16.93	0.44	0.44	
Salt	0.3		0.05	0.05	
Barley	22.85				
Soy hulls	8.04				
Molasses	2.98				
Mineral & vitamins calves	0.3				
Mineral & vitamins heifers		0.51	0.9	0.84	0.79

Abbreviations: AI: artificial insemination; DM: dry matter; DDG: distillers' corn grains.
